# Can social adversity alter the epigenome, trigger oral disease, and affect future generations?

**DOI:** 10.1007/s11845-024-03697-3

**Published:** 2024-05-14

**Authors:** Sakr Khalid, Michaela Kearney, David E. McReynolds

**Affiliations:** Dublin Dental University Hospital, Trinity College Dublin, Dublin, Ireland

**Keywords:** Allostatic load, Chromatin, Dental pulp, DNA methylation, DNA methyltransferase inhibitors, Epigenetic heredity, Epigenetics, Genetics, Health inequalities, Histone deacetylase inhibitors, Histone modification, Oral cancer, Oral disease, Periodontitis, Pulpitis, Social adversity, Stress

## Abstract

The nature versus nurture debate has intrigued scientific circles for decades. Although extensive research has established a clear relationship between genetics and disease development, recent evidence has highlighted the insufficiency of attributing adverse health outcomes to genetic factors alone. In fact, it has been suggested that environmental influences, such as socioeconomic position (SEP), may play a much larger role in the development of disease than previously thought, with extensive research suggesting that low SEP is associated with adverse health conditions. In relation to oral health, a higher prevalence of caries (tooth decay) exists among those of low SEP. Although little is known about the biological mechanisms underlying this relationship, epigenetic modifications resulting from environmental influences have been suggested to play an important role. This review explores the intersection of health inequalities and epigenetics, the role of early-life social adversity and its long-term epigenetic impacts, and how those living within the lower hierarchies of the socioeconomic pyramid are indeed at higher risk of developing diseases, particularly in relation to oral health. A deeper understanding of these mechanisms could lead to the development of targeted interventions for individuals of low SEP to improve oral health or identify those who are at higher risk of developing oral disease.

## Introduction

‘Nature or nurture?’ is a question that has been the subject of many scientific debates. Remarkably, geneticists can now precisely identify and locate certain genes in the DNA sequence that are associated with disease [1]. However, recent breakthroughs have established that the paradigm of thought that defines ‘nature’, or an individual’s DNA sequence, to be the sole determinant of disease outcome is insufficient. In fact, it has become apparent that other factors stemming from the environment and functioning above the genome may be at play [2]. In this regard, there is considerable interest in the field of epigenetics, which may explain the differences in health inequalities between individuals throughout the socioeconomic pyramid. The question of the relevance of early experiences of social adversity throughout life and how these impact biological processes has been at the forefront of epigenetic research over the past few decades.

In 1957, Conrad Hal Waddington, a developmental biologist, first put forward the ‘epigenetic landscape’, in which a ball could follow different paths as it rolls down a slope due to the roughness of the surface. The ball in this case represents the cell, whilst the slope represents cell differentiation. The variable roughness of the surface symbolises extracellular environmental factors which can influence the path the cell follows during differentiation. In other words, the ‘epigenetic landscape’ describes the process of cell differentiation during development. This explains how 200 different cell types can exist in the human body, with different functions, despite having the same DNA sequence [3]. Waddington later went on to define epigenetics as ‘the branch of biology which studies the causal interactions between genes and their products which bring the phenotype into being’ [4]. ‘Epi’ is a Greek prefix that means ‘above’, and so ‘epigenetics’ refers to factors functioning ‘above’ the genome. The epigenome, following signals from intra- and extra-cellular influences, results in modifications to the chromatin of the cell, which influences gene expression without causing alterations to the DNA sequence. In summary, it is a bridge between genotype and phenotype [4, 5].

## Review

### The genome and the epigenome

To understand how the epigenome links psychosocial stimuli to ‘get under the skin’ and become embedded into the genome to influence biological processes involved in diseases, a basic understanding of the structure of chromatin is required.

**Fig. 1:**
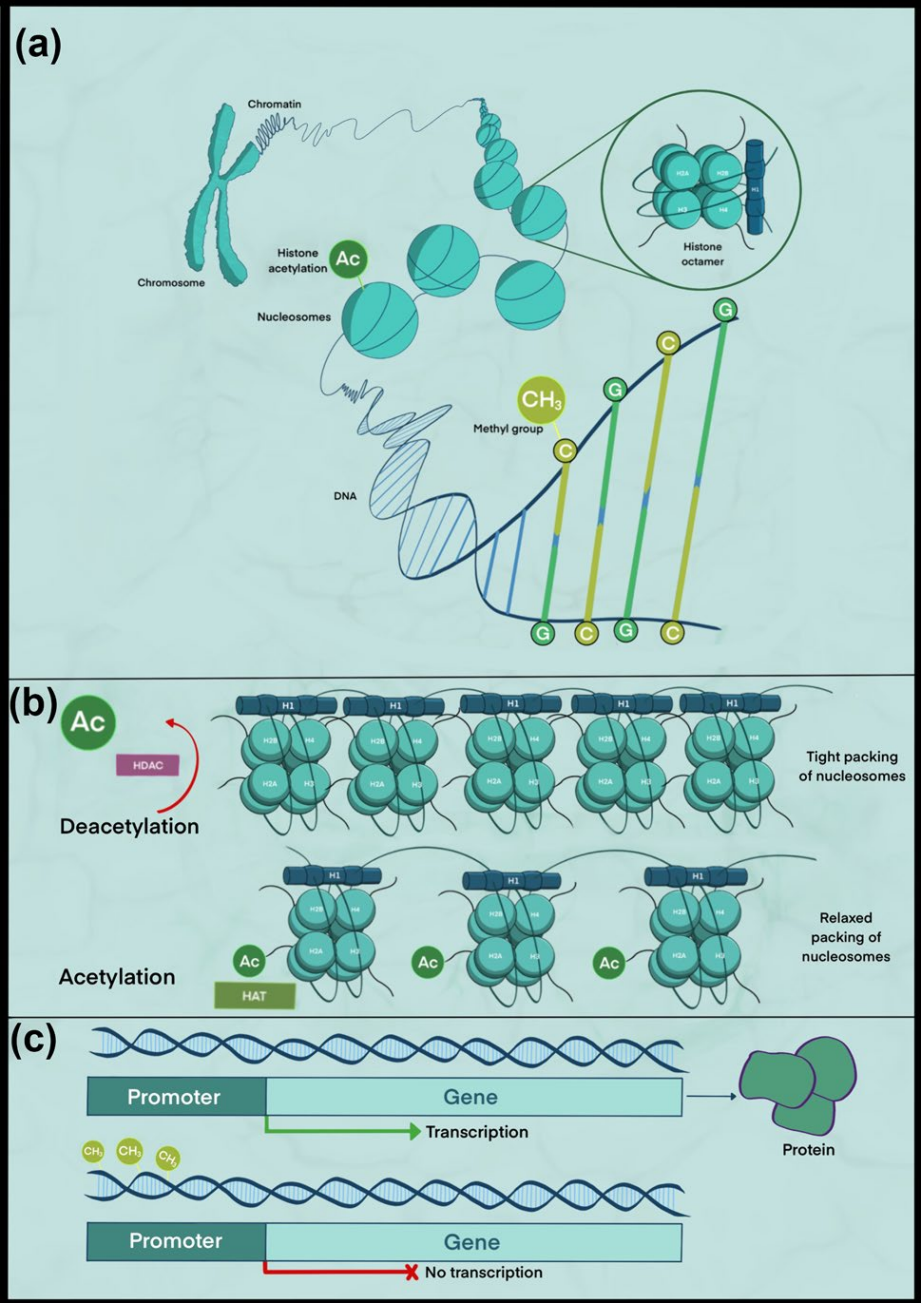
**a** Overview of chromatin organisation. Chromosomal DNA is tightly wrapped around histone proteins to form nucleosomes, which are further condensed into chromatin. Various modifications to the DNA and histones in this structure can stimulate or repress gene expression as required by the cell. **b** Histone acetylation. Acetyl groups can be added to or removed from histones to alter gene expression. This process is mediated by HATs and HDACs. **c** DNA methylation. Methyl groups are added to gene promoters in order to repress gene expression

Chromatin, a complex of DNA and protein found in all eukaryotic cells, consists of building blocks known as nucleosomes, each of which consists of 146 base pairs of nucleotides and one core histone complex, known as histone octamer [6–8]. Histones are involved in the tight packing of nucleosomes which enables 2 m of DNA to fit into the nucleus of every cell [9]. This packing formation also enables regulation of gene expression, as transcription factors are generally unable to bind to the condensed DNA, ultimately resulting in reduced production of proteins as required by the cell [10] (Fig. 1a). Epigenetic post-translational modifications (PTMs), most notably the addition of methyl or acetyl groups, take place around the N-terminal amino acid tails of the core histones [11, 12] and are capable of condensing or relaxing the nucleosome structure in a manner which affects gene expression.

The tight packing of nucleosomes that prevents gene expression can be induced by deacetylation, catalysed by a group of enzymes known as histone deacetylases (HDACs) which remove an acetyl group (−COCH_3_) from histone amino acid tails. In contrast, a relaxed chromatin conformation exposes transcription factor binding sites for gene expression and is mediated by the addition of an acetyl group to amino acid tails in the process of acetylation, catalysed by histone acetyltransferases (HATs) (Fig. 1b). The balance of histone acetylation and deacetylation is critical for normal cellular function, as evidenced by aberrant histone acetylation in many cancers [13]. Notably, extensive research has established histone deacetylase inhibitors (HDACi)—a class of synthetically produced biological agents—to have considerable therapeutic potential due to their ability to inhibit deacetylation [14, 15]. A number of HDACis have already been FDA-approved, such as suberoylanilide hydroxamic acid (SAHA) and panobinostat [16].

Methylation of histones is a process which involves the addition of a methyl group (–CH_**3**_) to lysine molecules on H3 and H4 core histones. This process most commonly occurs on H3 lysines K4 and K9, which results in gene expression and silencing, respectively. These chromatin modifications do not alter the DNA sequence itself, only its accessibility, resulting in potentially heritable alterations to gene expression [7].

The most studied epigenetic process is DNA methylation, which is modulated by enzymes known as DNMTs (DNA methyltransferases). This process involves the addition of a methyl group to a cytosine base to produce 5-methylcytosine (5mC) and typically occurs at regions of the genome with a high concentration of phosphate-linked cytosine-guanine dinucleotides (CpG islands) [17]. The methylated DNA recruits methyl-binding proteins that promote chromatin compaction and induce gene silencing through transcriptional repression [18] (Fig. 1c). Approximately 28 million CpG dinucleotides are present in the human genome, 60–80% of which are normally methylated as part of processes such as chromosome inactivation, heterochromatin folding, and the maintenance of genomic stability [19–21]. Loss of genomic stability through aberrant methylation of certain genes may result in the activation of oncogenesis, leading to the development of numerous cancers, including oral squamous cell carcinoma (OSCC) [22–24]. To counteract these cases of abnormal methylation, research in DNA methyltransferase inhibitors (DNMTi), another group of exciting biological agents that are likely to play a large role in cancer therapy in the foreseeable future, has been taking place. Their ability to inhibit DNMTs has proven to be successful in reversing abnormal DNA methylation associated with cancer cells [25]. At the time of writing, two drugs have been approved by the FDA and are currently in clinical use for the treatment of myelodysplastic syndrome and acute myeloid leukaemia [26].

### Epigenetic heredity

Certain mechanisms have been described to explain how epigenetic traits and modifications may be transmitted inter-generationally [27, 28]. It has been shown that immediately following fertilisation, the erasure of DNA methylation patterns of the gametes in the zygote occurs, which leads to the resetting of epigenetic marks between generations [29, 30]. However, neither DNA methylation nor histone modification patterns are fully erased between generations, which provides evidence of some potential inter-generational heritability [30]. It has also been shown that a large number of epigenetic marks also undergo reprogramming through demethylation during the meiosis of primordial germ cells, yet a substantial number of genes have been shown to retain the parental methylation patterns as a result of epigenetic transmission from primordial germ cells [31, 32].

In one study, rats exposed to endocrine disruptors demonstrated epigenetic modifications resulting in decreased male fertility through the male germline, extending from F1 to F4 generations [33]. In another study, mice subjected to specific odour fear-conditioning prior to conception later conceived F1 and F2 generations that demonstrated heightened sensitivity to that odour, despite having never been exposed to it [34].

It is not yet known how post-translational histone modifications are transmitted inter-generationally through cell mitosis [35]. However, following the disruption of nucleosomes ahead of replication, recombination of older parental histones with newer ones has been established, which may offer an insight into the method of epigenetic transmission [36], as parental histones may also modify and influence the new group of histones assembled [37].

### Adversity and long-lasting epigenetic impacts

One of the earliest records demonstrating how environmental conditions during mammalian development may induce epigenetic changes that last throughout life relates to a retrospective study carried out on adults who were prenatally exposed to famine during the Dutch Hunger Winter of 1944–1945 in the Netherlands [38]. In a landmark study, individuals who experienced prenatal famine exposure were found to carry distinct epigenetic marks six decades later. Specifically, the authors discovered hypomethylation of the *IGF2* (insulin-like growth factor 2) gene, which is a key factor for human growth and development, compared to same-sex siblings unexposed to prenatal malnutrition [39]. These adults who were exposed to famine during early gestation had a higher risk of developing diseases during late adulthood such as schizophrenia [40], generalised reduced cognitive ability [41], coronary artery disease [42], and obesity [43].

The role of social upbringing with regard to education and income has been shown to play a role in developing diseases throughout a person’s lifespan. The famous Whitehall study carried out first on British civil servants in 1967 demonstrated a strong inverse relationship between mortality and grade of employment across a seven-and-a-half-year follow-up. The researchers also found that men in the lower grade of employment were at higher risk of mortality specifically due to coronary heart disease (CHD). Even after controlling for other risk factors such as plasma cholesterol levels, systolic blood pressure (BP), cigarette consumption, and height, those with the lowest grade of employment still had a relative CHD risk of 2.1 compared to those with the highest grade of employment [44]. A follow-up longitudinal study conducted 20 years later—‘Whitehall II’—confirmed the inverse relationship between socioeconomic position and risk of CHD, metabolic syndrome, and diabetes [45].

### The theory of biological embodiment

The biopsychosocial model first introduced by Engel in 1979 argued that general health is a complex outcome of biological, psychological, and social factors [46, 47]. This was succeeded by a more recent theory known as ‘biological embodiment’; a concept which posits that complex processes due to social deprivation in its many forms bring about the initiation of pathobiological sequelae that leave a long-lasting mark on the biological systems in those vulnerable [48, 49]. One of the core concepts of how biological embodiment works is through ‘allostatic load’, or the preferred term, cumulative biological risk.

Allostasis refers to the body’s ability to achieve homeostasis or ‘stability’ through internal physiological or behavioural changes [48], whilst allostatic load (AL) refers to the ‘cumulative wear and tear on body systems as a result of exposure to chronic stress’, where a person’s inability to adapt emotionally and physiologically to increasingly stressful demands results in a compounded dysregulation over time. The risk of disease occurrence is higher in such cases, resulting from a combination of excessive cortisol release and dysregulation of mechanisms that shut down cortisol release through negative feedback mechanisms once the stressor has resolved [50]. Not only does AL result in increased cardiovascular, metabolic and general physical decline, and all-cause mortality [51], but it has also been linked to alterations in brain function, which is the key organ of stress processes [52–55]. AL can be scored through biometric parameters such as BMI (body mass index), waist-to-hip ratio, resting systolic and diastolic BP, serum high-density lipoprotein, glycated haemoglobin, and C-reactive protein alongside others, all of which have been shown to be socially patterned and elevated in those of lower SEP [56–58].

### The role of stress in influencing the epigenome

Factors such as stress [59], diet [60], infection [61], smoking [62], maternal care during infancy [63], and even increasing age [64] may influence epigenetic modifications to alter the chromatin state and expose certain genome stretches for longer or shorter periods, subsequently resulting in variability and dysregulation of protein production. Stress, in particular, may play a detrimental role in the dysregulation of the hippocampus-pituitary-adrenal (HPA) axis [65, 66] and the sympathetic-adrenal-medullary (SAM) pathway [67]. The activation of the HPA axis due to chronic stress results in the release of cortisol into the blood via a complex signalling cascade [68], whilst the SAM pathway is activated by the stimulation of the adrenal medulla to release the catecholamines epinephrine and norepinephrine [67].

Repeated and chronic exposure to cortisol has numerous pathological sequelae, such as increased BP, reduced metabolism, and subsequent risk of obesity, alongside chronic anxiety and depression [69]. Chronic exposure to stress has numerous immunosuppressive effects, which include depression of proliferation and differentiation of immune cells (which have receptors for cortisol and epinephrine/norepinephrine) [70] and disruption of pro-inflammatory cytokine production by directly binding to immune cell surfaces at specific sites, resulting in anti-inflammatory effects throughout the body [71]. Elevated levels of cortisol have also been directly linked with increased production of pro-inflammatory cytokines, which are conducive to periodontal tissue destruction, and an increased count of active periodontal pockets in those suffering from periodontitis [72–75].

In contrast, acute episodes of stress trigger the release of norepinephrine through the SAM pathway, which binds to its receptors at cells in lymphoid organs to modulate immune cell function [67], leading to decreased blood flow to periodontal tissue, hence reducing the diffusion of cells and nutrients, resulting in poor wound healing throughout the periodontium [76, 77].

Paradoxically, individuals who are chronically stressed have been associated with increased inflammation, despite the anti-inflammatory functions of cortisol as mentioned earlier. This is due to the desensitisation of the glucocorticoid receptor pathway following long-term exposure to cortisol, rendering the immune cells insensitive to its anti-inflammatory effects [78]. Further studies have attributed this to epigenetic processes that have numerous effects including suppression of the *NR3C1* gene (glucocorticoid receptor gene) [79]. Stressful experiences in early life result in disruptions to the *NR3C1* gene in the hippocampus through DNA methylation, resulting in long-lasting and reduced glucocorticoid expression and HPA axis dysfunction. This low expression is linked with insufficient negative feedback mechanisms that keep cortisol in circulation for prolonged periods and result in chronic high-stress states [80–82].

Indeed, studies have demonstrated that under conditions of social isolation [83, 84] and low SEP [85, 86], there is an alteration to the HPA axis regulatory function. Studies have also shed light on the resultant association with reduced leukocyte sensitivity to glucocorticoid regulation [87, 88], leading to increased risk of disease susceptibility [89].

### Epigenetics and oral health

It has already been established that oral diseases such as caries [90] and periodontitis [91] disproportionately affect those who are more socially disadvantaged and who occupy lower socio-economic positions. Whilst this is generally attributed to lifestyle factors associated with these groups, such as poor oral hygiene [92], increased sugar consumption [93], and high rates of smoking [94] and alcohol consumption [95], more evidence is shedding light on the long-term epigenetic effects associated with low SES during critical stages of fetal development and early childhood. Exposure to the associated social and environmental stresses leads to their embedding and imprinting into the genome at an early age, resulting in long-term cascades of side effects that place these individuals at higher risk of developing oral diseases. These experiences of early stressful events and their long-term effects on brain health and mental well-being are well documented [96–99]. In accordance with this model, a recent study found structural changes in the enamel of primary teeth in children who had experienced perinatal maternal psychosocial stress [100]. In another pertinent study, accelerated eruption of primary molars in children of lower-income families was demonstrated [101], aligning with a growing body of evidence in the literature linking experiences of early psychosocial stress to accelerated epigenetic aging [102].

Furthermore, there is compelling evidence suggesting a variety of oral microbiomes that are more consistent with varying socioeconomic groups than oral health behaviours and more consistent with certain ethnic and racial groups than diet [103]. Interestingly, elevated levels of cortisol in children with lower SEP demonstrated higher levels of the cariogenic bacteria *Streptococcus mutans* in the oral cavity [104], adding another link to the complex cariogenic mechanisms involved in children with lower SEP who have already been shown to be at higher risk of developing more severe forms of dental caries and tooth loss [105–107]. Another study found higher levels of IgG antibodies against *Bacteroides forysthus* bacteria, which is associated with periodontal disease, in groups with higher levels of depression and psychosocial stress [108].

In the context of periodontitis at the molecular level, a significant finding emerged regarding the epigenome of an individual suffering from the inflammatory disease, where different methylation patterns were observed between inflamed and non-inflamed sites [109]. It has also been shown that *TNFA* and *COX2* CpG sites are hypermethylated and silenced in chronic periodontitis [110, 111], whilst in another study, hypomethylation at the promoter region of *IFNY* was demonstrated, which led to increased expression of IFN-γ in biopsies from tissues with periodontitis in comparison to tissues of a healthy periodontium [112]. IFN-γ cytokines have been linked to pulpal inflammation, with the *IFNY* promoter region demonstrated to be only partially methylated or unmethylated in 94% of inflamed pulp tissue samples, compared with healthy pulp tissue samples, of which 44% demonstrated total methylation of the gene [113]. The same authors conducted a separate study exploring the differences between methylation patterns of the *TLR2* (Toll-like receptor 2) gene and *CD14* (a TLR4 co-receptor) gene in inflamed and non-inflamed pulps and found no difference in methylation patterns between the two groups [114]. These findings emphasise the novelty and complexity of the research being carried out concerning epigenetic mechanisms and their dental implications and present new insights that may revolutionise our treatment of pulpitis.

In another study, hypermethylation and silencing of the *TLR2* gene in tissues with periodontitis were confirmed, and the authors found a correlation between the methylation status of the *TLR2* gene and probing depths [115]. Additionally, the gene encoding the IL-8 cytokine, which plays critical roles in the recruitment of neutrophils to inflamed periodontal sites, was found to be hypomethylated at the promoter region in epithelial cells of those with periodontitis [116] and aggressive periodontitis [117] compared to healthy individuals, resulting in increased expression in those suffering from periodontal disease. These findings offer a glimpse into the complexity of mechanisms underlying periodontitis and the dysregulation of genes associated with inflammation and immune response in the context of this oral condition. Epigenetic alterations influencing the inflammatory response may not be permanent. Hence, by altering the oral microbiome that was conducive to periodontal disease initially, for example, through deep cleaning of pockets, the epigenome of these inflamed sites may be reset.

Drawing parallels to tumour development, it was demonstrated that the CpG islands of tumour suppressor genes, such as *P16*, are often hypermethylated at the transcriptional promoter region leading to gene silencing in oral premalignant lesions, making it a potential predictive marker of the risk of malignant change in oral epithelial dysplasia [118]. Positive correlations between smoking and hypermethylation at the promoter region of *P16* in patients with non-small cell lung carcinoma were demonstrated [119], whilst another study showed that consumption of nicotine-derived nitrosamine ketone in tobacco is associated with hypermethylation of the *P16* gene promoter [120]. A prospective cohort study investigated the link between the methylation status of the *P16* promoter region and the progression of oral epithelial dysplasia over a period of 46 months. Patients with *P16*-methylated dysplasia were found to be at a significantly higher risk of developing oral cancer than the control group of patients with *P16*-unmethylated dysplasia [121]. Interestingly, one study found that treatment with procaine, a DNMTi, reversed the hypermethylation of the *PAX9* gene, which is partly involved in the differentiation of OSCC cells. The reversal of methylation and increased expression of *PAX9* induced apoptosis and cell growth inhibition in OSCC tissue cells *in vivo* and *in vitro* and improved anticancer drug sensitivity [122], highlighting the potential role of DNMTi drugs in future oral cancer treatment.

There have been a limited number of studies linking histone modification to periodontal disease and pulpal inflammatory processes [123–126]; however, in relation to other inflammatory diseases, aberrant histone modification processes have been demonstrated to be involved in rheumatoid arthritis [127], diabetes mellitus [128], and asthma [129]. In addition, whilst a role for histone modifications in periodontal and pulpal inflammation has yet to be elucidated, one study found that treatment of mice with periodontitis with a HDACi increased bone volume *in vivo*, suggesting a potential role for HDACi in ameliorating the adverse outcomes of periodontal disease [124].

In addition to their potential therapeutic use as anti-inflammatory drugs, *in vitro* [130] and *in vivo* [131] studies have demonstrated their potential as therapeutic agents for the treatment of cancers due to their anti-tumourigenic effects in transformed cells, including growth arrest and autophagic cell death, whilst normal cells are more resistant to these changes [132–134]. At the time of writing, four HDACi drugs are approved by the FDA for the clinical treatment of certain tumours [135]. With regards to non-oncological normal tissue, there is great therapeutic potential for the use of HDACi and DNMTi in stimulating pulpal tissue regeneration as part of restorative dental treatment. The increased appreciation for epigenetic inhibitors in this regard is in large part due to their ability to modulate a number of complex processes which are crucial for dentinogenesis, including inflammation, mineralisation, and stem cell differentiation [136, 137].

Epigenetic mechanisms involving histone demethylases (HDMs) and histone acetyltransferases (HATs) [138] have also been shown to play crucial roles at certain stages of tooth development and may be linked to the induction of dental anomalies such as supernumeraries and hypodontia. This has been demonstrated in studies involving mice and monozygotic human twins who share a similar genetic makeup but demonstrate different dental phenotypes owing to certain environmental influences at certain stages during tooth development [139, 140].

## Conclusion

Based on the current literature, evidence suggests that social adversity, particularly when experienced at an early age, plays a significant role in altering the epigenome in numerous ways, through various mechanisms. The literature describing the role of epigenetics in oral disease and dental anomalies is still in its infancy, yet there is a significant body of evidence linking psychosocial stress to many endocrine and inflammatory diseases, particularly periodontitis and oral cancer. Some of the alterations to the genome have been shown to be long-lasting with an ability to be passed down inter-generationally, at least when demonstrated through *in vitro* and *in vivo* studies, not involving humans. However, the mechanisms of epigenetic heredity are yet to be fully explained. A more comprehensive understanding of these molecular processes may enable us, through targeted interventions, to influence the genome to reduce disease risk or identify individuals who are at an increased risk of developing oral diseases.
